# Do Competitive Contexts Affect Mindreading Performance?

**DOI:** 10.3389/fpsyg.2020.01284

**Published:** 2020-06-23

**Authors:** Livia Colle, Giancarlo Dimaggio, Antonino Carcione, Giuseppe Nicolò, Antonio Semerari, Claudia Chiavarino

**Affiliations:** ^1^Department of Psychology, University of Turin, Turin, Italy; ^2^III Centro Psicoterapia Cognitiva, Rome, Italy; ^3^Centro di Terapia Metacognitiva Interpersonale (CTMI), Rome, Italy; ^4^Istituto Universitario Salesiano Torino Rebaudengo (IUSTO), Turin, Italy

**Keywords:** mindreading/theory of mind, metacognition, competition, social rank, personality traits

## Abstract

Mindreading is contingent upon interpersonal context. Little is known about how competitive contexts influence mindreading skills. The idea was that the capacity to think about mental states would decline when individuals experiencing failure in competition. This study aims to assess effects of a competitive experience (a computer competitive PC game) on a sample of healthy subjects (119 participants). The sample was divided into two sub-samples. The experimental group underwent an experience of failure, consisting in a PC game of logic against a hypothetical opponent. The control group was required instead only to discuss past personal experiences of competitive interactions. The Metacognitive Assessment Interview was administered to each sub-sample for evaluating mindreading capacities. Self-report tests were additionally provided for evaluation of trait-based dispositions: self-esteem, perfectionism, narcissism. Results supported our hypothesis: induction of sense of failure compromises ability to describe one’s own mental states and mental states of others. This effect was more pronounced in the domain of self-reflection. Results remained significant after controlling for self-esteem, perfectionism, and narcissism. We discuss possible clinical implications of these findings and the importance of evaluating mindreading capacities under the pressure of social rank as well as of other social motive.

## Introduction

When humans are under pressure of competition, they have increased need to be aware of their inner experiences and to make accurate guesses about the mental states of others. They need to recognize if they are afraid of losing, if they still have resources, if the task is important enough or, alternatively, if they are devoting too much time and effort to a task that is less relevant than it seems. At the same time, they need to form an accurate and possibly realistic picture of what is passing through the mind of the others. Being aware of one’s own feelings and what has elicited them helps decision-making, while realizing what is passing through the mind of others and the motive driving overt behavior is necessary for adjusting own action so as to maximize the chances that one’s own personal goal will be fulfilled and that relationships will be as smooth and stable as possible.

Some authors have noted that mindreading is relevant in the competition context (Humphrey, 1976; Gergely and Unoka, 2008; Liotti and Gilbert, 2011). Chimpanzees for example appear to have some form of understanding of the intentions of the others in the competition context (Cheney et al., 1986). Conversely, chimpanzees do not demonstrate the same level of intentions’ understanding intentionality in situations where a human partner proposes a cooperative interaction (Warneken et al., 2006).

Unfortunately, the pressure of competition may narrow our capacity to make sense of mental states, so we may have limited abilities in the very moment we need them. Fear of failure may steer our attention to signals of incapacity in the self or of criticism in the others. Shame about feeling inferior may let us forget the many moments in which we succeeded, or simply discard relevant needs, such as relaxing, searching for social sharing, and playing. Under the background of these reflections, what do we currently know about the effects of competition on capacities to make sense of mental states?

### Mindreading

The human capacity to recognize and reflect upon mental states has been variously defined as mindreading, mentalization, metacognition, and theory of mind (Baron-Cohen, 1995; Semerari et al., 2003; Bateman and Fonagy, 2004).

Being aware of the differences, we henceforth use the term mindreading because it encompasses different domains of awareness and reflection on both one’s own and others mental states. In this study we use the term mindreading as it is possibly the more inclusive term referring to the capacity for mentalistic reasoning. This capacity includes: naming and distinguishing the feeling one is experiencing, describing the complexity of one’s own thoughts in the situation. It also includes the awareness that one’s ideas are open to question and do not necessarily reflect reality. Another aspect is the capacity to understand what the others think and feel and what drives their behavior on the basis of cues, such as facial expressions, behavior, and personal history (Semerari et al., 2003; Dimaggio and Lysaker, 2015).

Poor mindreading may manifest itself under specific types of social interaction experienced as problematic. As we earlier observed, mindreading is particularly necessary when humans are in the heat of personally relevant interactions. As an example, a child driven by attachment needs to express distress in a way that will move the caregiver to care of her. Clinical observations made the point that in persons with some psychiatric disorders, mindreading is compromised exactly when it is most needed, that is in the heat of interactions driven by primary social needs, such as attachment. These persons actually get confused about what they feel and form negative and rigid representations, about intentions, thoughts, and feelings of the others (Semerari et al., 2005, 2015; Choi-Kain and Gunderson, 2008), with the final result being a problematic interaction filled with tension, conflict and neglect (Clarkin et al., 1999; American Psychiatric Association, 2013).

Understanding under what conditions mindreading capacities are compromised is relevant in order to know how to protect individuals against such a decline or in order to predict where this momentary collapse will happen and deal with it. Historically, the major focus has been on the relationship between activation of attachment and decline in mindreading in a series of psychiatric condition, ranging from borderline personality disorders to post-traumatic disorders and psychosis (Fonagy and Bateman, 2007; Liotti and Gumley, 2008; Fonagy et al., 2011; Sharp et al., 2012). Supporting clinical observations, research has noted that persons having histories of disturbed attachment, in particular disorganized, experience severe decline in the capacity to understand and integrate mental states (Fonagy, 1991; Liotti, 2004, 2006).

In the clinical fields some authors have noted that mindreading may not just be compromised in the context of disturbed attachment. Other evolutionary selected motives may be at the roots of state-dependent failure in making sense of mental states (Dimaggio et al., 2007, 2015b; Fonagy and Luyten, 2009; Liotti and Gilbert, 2011; Dimaggio and Lysaker, 2015) noted that this capacity collapses under the activation of disturbed attachment, which diminishes the possibility that persons with some pathology, e.g., borderline personality disorder, will feel nurtured and protected by others.

### Social Rank and Mindreading

Among these motives, social rank, that is competition or antagonism, seems to be a key factor underlying momentary mindreading failures. Humans are continuously facing competitive contexts, driven by social rank (Gilbert, 1989, 2005). Examples are doing a university exam, playing a sport match and so on. There are persons with some trait-based tendencies who are more sensitive to social rank issues. Persons low in self-esteem for example may be over-sensitive to threats to their image (Gilbert et al., 2002). Highly perfectionistic individuals may be over-sensitive to rank as well: they are over concerned about their performance, a measure of their personal worth, and are concerned about own mistakes and flaws and are afraid of criticism (Hewitt and Flett, 1991). Also individuals high in narcissism are hypersensitive to status threats (Mahadevan et al., 2018). Overall, it might well be that persons low (self-esteem) or high (perfectionism and narcissism) in these traits may be prone to mindreading impairments when they have to focus on experiences of defeat or failure.

The influence of competition and social rank on mindreading has undergone some research scrutiny. First, establishing social rank is relevant, as humans need to know the positions others occupy in the hierarchy. Actually, individuals have a bias toward faster attribution of rank when they think the other is above them, and then their defense system is more quickly turned on as a response to possible threats (Haaker et al., 2016). Zink et al. (2008) found that when presented with an unstable hierarchy during a game, that is they had to understand the rank position of the others, individuals activated more brain areas supporting social cognition, than when the hierarchy was stable. Mindreading may vary according to different motives underlying a social exchange. Recent research showed that taking into account the specific different psychological features attributed to the agent or to the responder is influential in economic decision and define much to reciprocate in the Ultimatum Game (in other words their mind-reading) (Rabin, 1993; Dufwenberg and Kirchsteiger, 2004). A key question for reciprocate in economic decision is how agents evaluate the kindness of a particular action (Stanca et al., 2009). Agents decide for rewarding kind actions and punishing unkind actions, even if this is costly in terms of material payoffs depending on the perceived kindness of other’s action. In other words, they decide according to their ideas about the other agents’ intentions. In these models, actions with identical outcomes may elicit different reciprocating responses depending on how each partner interpret his/her opponent’s social intention, e.g., whether he/she is cooperative vs. competitive (Iannello and Antonietti, 2008).

In fact, Lee et al. (2018) noted that performance when playing Tetris was better under cooperation than under competition. Moreover, under cooperation brain areas related to mindreading were more active. In a second part of the experiment pictures of the others in pain were displayed. In the context of cooperation individuals displayed more empathy then in the context of competition, though only for participants with higher baseline empathic tendencies. Overall results suggested that competition both decreased performance and shut down mindreading. Others have noted than cooperation activates mindreading regions in the brain, such as the dorsomedial prefrontal cortex (dmPFC), temporoparietal junction (TPJ), and superior temporal sulcus (McCabe et al., 2001; Rilling et al., 2002; Decety and Jackson, 2004; King-Casas et al., 2005; Elliot et al., 2006). In contrast, competition activates inference-related brain regions and similar mindreading regions (Lee et al., 2018), mostly for the sake to understanding the mental state of the competitors and so as to maximize the odds of receiving the incentive (Halko et al., 2009). As Lee et al. (2018) noted, cooperation and competition activated mindreading for different purposes, though their experiment suggests that eventually cooperation is related to better social cognitive functioning. This is consistent with Tomasello (2008) observations that cooperation has evolved as a mechanisms involving shared attention and mutual understanding of mental states so as to the maximize the likelihood that a group may access to resources.

Consistently, Falco et al. (2019), found than participants playing the Ultimatum Game made different decisions when they thought their opponents were making an offer because they included and attributed to them higher social rank, then when they considered the offer of the others as generated by devaluing. In other words, when moved by competition participants accepted fewer unfair offers motivated by a negative view of a rival. When moved by social inclusion instead they accepted more unfair offers as they thought they were about to be included in a group.

Attention to the link between mindreading and social rank has been given by clinicians as well. Gilbert (2000) noted how humans engage in complex activities for monitoring the relative strengths/competencies of self in relation to others, and the skills and intentions of others, so that the “weaker” disengage from, and rarely instigate conflict with, the “stronger” (Gilbert, 2014, p. 8). Liotti and Gilbert (2011) noted how humans highly sensitive to social rank issue may steer mentalizing for the purpose of submission so to appease the other. Following Gilbert, others have noted the possible connection between social rank and reduced mindreading capacities both in oneself and in others (Dimaggio et al., 2015a; Colle et al., 2017; Popolo et al., 2019). Monticelli et al. (2018) found that patients in psychotherapy showed lower levels of mindreading when social rank was active during sessions.

### Social Rank and Mindreading; What We Do Not Yet Know

The connections between activation of the social rank motive and variations in mindreading have been hypothesized and investigated. Research have highlighted that social rank may bias our perceptions of others and that under the activation of this social motive capacities for understanding mental states may be narrower than when other motives (e.g., cooperation) are active (Liotti and Gilbert, 2011; Lee et al., 2018). There are issues still needing to be sorted out.

First, studies focus on how individuals make sense of the states of mind of the others. To the best of our knowledge, there is little into how social rank affects understanding of one’s own inner states. Another issue is that research has mostly been performed using behavioral (e.g., choices during games) and neuroimage measures of mental states. What is missing is an assessment of mindreading capacities as emerging from the individual’s subjective experience. In other words, how does the immediacy of competitive interactions affect the way subjects attribute mental states to themselves and to each other. Moreover, it is necessary to measure mindreading in real interactions among individuals, rather than solely in the laboratory. This *second-person* approach emphasizes that experiencing and interacting with others are crucial primary ways of evaluating levels of mindreading. Recent studies have also drawn attention to the fact that tasks which involve engagement in direct personal interactions may affect mindreading more than off line tasks (which are similar to remembered past experience of failure, Schilbach, 2016).

Our research question was: when primed with a competitive situation where they were convinced they failed, will individuals have reduced general capacities to understand their own mental states and those of others? Specifically, we assessed mindreading in two different situations: an online situation where participants were directly involved in a game focused on winning or losing; an indirect situation where participants were asked to describe the mental states during a past experience of competition. We therefore focused on analysis of discourse as emerging in a semi-structured interview where the object of the reflections was not the mental state of the opponent in a game, but the overall capacity to ascribe mental states to self and others.

### Hypothesis

Our hypothesis was that when primed with the idea of having failed a task where they faced a more successful opponent, individuals would display reduced capacity for mindreading both as regarding both self and others. We expected to find significantly lower mindreading levels in the group primed with an experience of failure than in the control group who did not participate in the competitive induction.

We also considered that some trait-based dispositions – in particular self-esteem, perfectionism, narcissism – may make some subgroups more prone to the effect of social rank, so we controlled for their effect on mindreading. Specifically, we controlled whether persons low in self-esteem, high in perfectionism, or high in narcissism can have a more pronounced impact on experiences of failures on their mindreading capacity.

## Materials and Methods

### Participants

A sample of 136 individuals from the population of Psychology students resident in Turin and surrounding areas were administered a series of self-report tests on a voluntary basis. Of this initial sample, 119 participants (65 men and 54 women) were available for the second phase of the test. A pseudo-random matching procedure was used for the allocation of the participants to the groups. First, couples of participants were formed that were matched on sex, age, and education. Then, within each pair, participants were randomly allocated to the experimental or to the control group. The average age of the sample was 26.2 ± 8.4 years and the age range was 18–63 years.

### Experimental Procedures

In a first session of the experiment, participants were recruited among the student population of Turin by last year students working for their final project. Those who accepted to participate were lead to a quiet room and asked to complete a series of paper and pencil self-administered questionnaires, which tested self-esteem, perfectionism, and grandiose and vulnerable narcissistic traits. The sample was then divided into two sub-samples matched by gender, age, and education.

In a second session of the experiment about 1 month later, the participants were called back to a laboratory room of the University, in order to complete one of two experimental conditions: induction of an experience of failure (experimental group, *n* = 66) and recall of past experiences of failing a competition (control group, *n* = 53). In order to evoke a sense of failure, we created a tweaked version of a very common game, known as “Minesweepers” or “Flowery Meadows,” involving logical and deductive skills. The aim of the game is to open the highest possible number of “mine-free” cells (mines are concealed), while taking into account the numbers which flash up on the clicked cells as one proceeds. These numbers represent the number of mines still concealed in the eight cells adjacent to the flashing number. In our version, it was impossible to win the task. Our goal was to induce a state of distressing arousal in the participants of the experimental group, as a consequence of losing the game.

Participants were informed that they would be playing an online game against another player. They were provided with the false information that they and their opponent had been matched on the basis of their own self-assessment of computer game expertise, as stated in the questionnaire administered during the first phase of the experiment.

**FIGURE 1:**
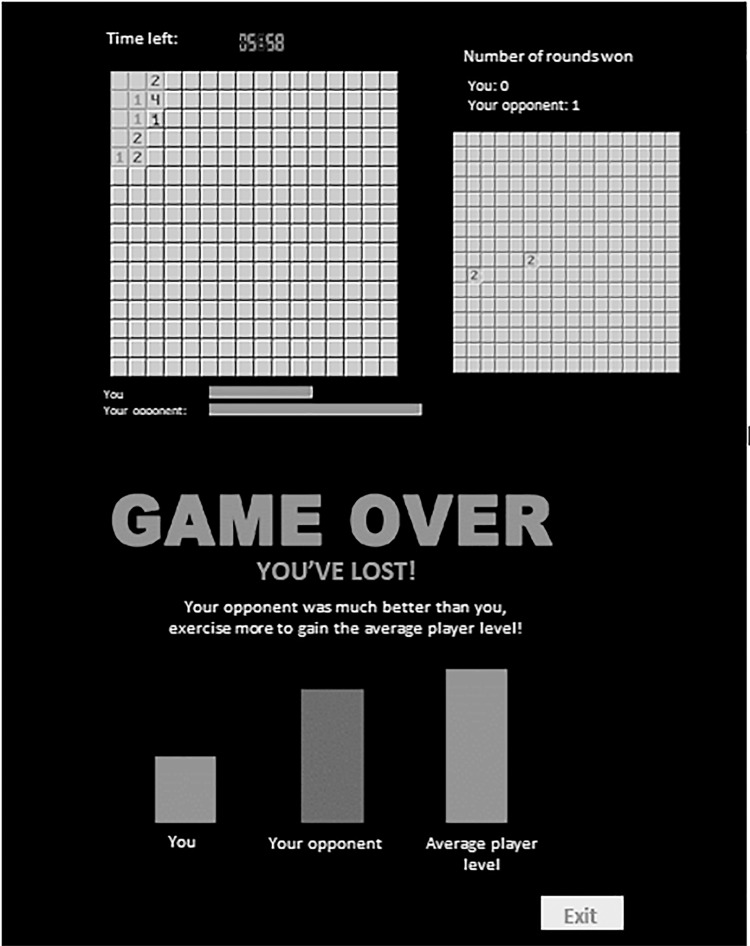
Screenshot of the tweaked version of Minesweepers game.

Participants simultaneously viewed two screens: the larger one displaying their own game, and the smaller one showing the opponent’s game in progress. The game software was modified so that the final score of the participant would always equal half the points scored by the virtual opponent. In order to emphasize the player’s defeat, a number of feedback messages appeared on the screen during the 10-min game. These pop-ups indicated the scores of “both” players. When time was up, two different screen pictures appeared. The first was a notice: “GAME OVER, you lost!” and the second was a bar graph showing the points scored by the participant, the score of the opponent (around twice as many points), and the average score of players at the same level of computer game expertise as the participant. This last bar displayed slightly higher levels than that of the opponent, creating the impression that one had lost against an average adversary and not against a top-player (Figure 1).

Finally, at the end of this second session, the Metacognitive Assessment Interview was administered. The participants of the experimental group who had undergone the induction, that is the experience of losing the game, were interviewed with the MAI immediately after the game, and the interview made specific reference to their subjective experience during the task. The control group did not play any game and was simply asked to talk about past experiences of failing a competition.

### Measures

*Metacognition Assessment Interview* (MAI; Semerari et al., 2012; Pellecchia et al., 2015): it is a semi-structured clinical interview constructed around a narrative task and designed to elicit and evaluate the metacognitive abilities of participants. Participants are asked to recall and give a brief account of a psychologically meaningful experience or event situated during the previous 6 months. The reported experience must be autobiographical and must involve at least one other person, so that interviewers can evaluate participants’ ability to understand the mental states of others. Once this brief narrative task is completed, participants are asked to answer a series of specific questions based upon the account and designed to evaluate four metacognitive sub-functions: *Monitoring*, *Integration*, *Differentiation*, and *Decentration*.

*Monitoring* refers to the ability to identify and label aspects of mental states: emotions, thoughts, motivations, and desires.

*Integration* refers to the ability to hold a coherent picture of self and other states. It includes the capacity to realize one may experience different states under different circumstances but still maintaining a sense of coherence.

*Differentiation* refers to the ability to realize that one’s own ideas do not necessarily mirror external reality, thus recognizing that they are subjective and may change when things are seen from a different perspective.

*Decentration* refers to the ability to adopt the perspectives of others and to make plausible hypotheses about their mental states. This is a form of perspective-taking similar to the concept of decentration described by Piaget and Inhelder (1970) and is keen to the concept of allocentrism (Frith and de Vignemont, 2005). Individuals with poor decentration ability find it difficult to grasp other people’s point of view rather than from their own standpoint.

The MAI inter-rater reliability was tested in a previous study, with Intraclass Correlation Coefficients of the different MAI functions ranging from 0.45 to 0.78 (all *p* < 0.001) (Pellecchia et al., 2015).

*Rosenberg Self-Esteem Scale* (SES; Rosenberg, 1965): it is a 10-item self-report questionnaire that measures global, explicit self-esteem. It is rated on a four-point scale ranging from 1 = “strongly agree” to 4 = “strongly disagree” (total score range: 10–40); higher scores indicate higher self-esteem. Previous studies have reported alpha reliabilities for the RSE ranging from 0.72 to 0.88 (Gray-Little et al., 1997).

*Multidimensional Perfectionism Scale* (*MPS*; Frost et al., 1990): it is a self-report questionnaire composed of 35 items grouped into 6 sub-scales: personal objectives, fear of making mistakes, parental criticism, parental expectations, doubts about one’s own actions, and organization. The MPS is rated on a five-point scale ranging from 1 = “not at all” to 5 = “extremely” (total score range: 35–175). The reliability of the Italian version of the scale, measured using Cronbach’s alpha coefficient, is higher than 0.75 for all the sub-scales (Lombardo, 2008). In this study we used the sum-score of scales 2, 3, and 5, which grouped together describe the core of maladaptive perfectionism (score range: 17–85).

*Narcissistic Personality Inventory* (*NPI*; Raskin and Terry, 1988): it is a self-report questionnaire which provides an index of narcissism reflecting both pathological levels as well as less extreme forms of narcissism that are believed to reflect narcissism as a personality trait. It is made of 40 alternative choice options, with opt-out not permitted (total score range: 0–40). In each item, one response is considered as indicative of narcissism and the other is not. Higher scores indicate higher levels of grandiose narcissism. The NPI total scale has been found to possess adequate internal consistency (alphas ranging from 0.82 to 0.84; Raskin and Terry, 1988).

*Hypersensitive Narcissism Scale* (*HSNS*; Hendin and Cheek, 1997): it is a 10-item self-report measure that dimensionally assesses hypersensitive narcissism. It is rated on a five-point scale ranging from 1 = “very uncharacteristic” to 5 = “very characteristic” (total score range: 10–50). The items capture the characteristic sensitivities of vulnerable narcissism. The reliability of the Italian version of the scale in a non-clinical sample, measured using Cronbach’s alpha coefficient, was 0.69 (Fossati et al., 2009).

An additional question regarding computer games was added to the baseline questionnaires: whether the participants played computer games, and, if so, how frequently and which were their favorites. They were also asked to provide a self-assessment of their personal expertise in each of their favorite games. These questions had the purpose to enhance the credibility of the competition for the participants in the experimental group.

### Statistical Analyses

To test for our hypothesis, correlation analyses were first conducted to assess the relation among the variables. Three hierarchical multiple linear regression analyses were then performed with total score of the MAI, Self score, and Other score as the dependent variables. In each of these three analyses, the baseline psychological variables (self-esteem, perfectionism, grandiose and vulnerable narcissism), were entered together in the first block of the analysis to control for their effect on mindreading, and participation in the competitive induction procedure (experimental group, control group) was entered in the second block. All the analyses were performed with SPSS 20.0 (SPSS Inc., Chicago, IL, United States).

## Results

Correlation analyses showed that participating in the induction procedure was associated to lower total scores and Self scores of the MAI. As regard correlations of MAI and underlying traits, grandiose narcissism as assessed with the NPI was associated to lower mindreading, both the Total score and Self and Others sub-scales. Mindreading was not associated with any of the other traits, that is Self Esteem (SES), perfectionism (MPS), and Vulnerable Narcissism (HSNS) (Tables 1, 2).

**TABLE 1:** Correlation coefficients among mindreading (MAI total score, MAI Self score, MAI Other score), trait-based dispositions (SES, MPS, NPI, HSNS), and participation in the Induction procedure.

Variable	2	3	4	5	6	7	8
1. MAI_tot	0.905**	0.868**	0.037	0.007	−0.300**	−0.095	−0.221*
2. MAI_Self		0.574**	0.018	0.054	−0.220*	−0.066	−0.301**
3. MAI_Other			0.105	−0.096	−0.296**	−0.095	−0.125
4. SES				−0.160	0.333**	−0.198*	−0.160
5. MPS					0.141	0.367**	−0.025
6. NPI						0.234*	−0.061
7. HSNS							−0.139
8. Induction^§^							

MAI, Metacognition Assessment Interview; SES, Rosenberg Self-Esteem Scale; MPS, Multidimensional Perfectionism Scale; NPI, Narcissistic Personality Inventory; HSNS, Hypersensitive Narcissism Scale. *p < 0.05, **p < 0.01; ^§^ 0 = no, 1 = yes.

**TABLE 2:** Descriptive statistics (Min-Max, Mean, and SD) of the variables tested in the study.

Variable	Min-Max	Mean	*SD*
MAI_tot	13–30	22.8	4.1
MAI_Self	5–15	11.2	2.5
MAI_Other	7–15	11.7	2.1
SES	11–33	22.8	4.6
MPS	17–136	41.3	3.0
NPI	1–28	13.6	5.7
HSNS	12–41	27.6	5.5

*MAI, Metacognition Assessment Interview; SES, Rosenberg Self-Esteem Scale; MPS, Multidimensional Perfectionism Scale; NPI, Narcissistic Personality Inventory; HSNS, Hypersensitive Narcissism Scale.*

Then, a hierarchical multiple linear regression analysis was performed with the MAI total score as the dependent variable. The trait-like control variables (self-esteem, perfectionism, grandiose, and vulnerable narcissism), entered in the first block, accounted for 11.9% of the variance of mindreading, with grandiose narcissistic traits showing the greatest effect. Consistent with the hypothesis, the induction accounted for an additional 5.0% of the variance of mindreading after controlling for all the baseline variables (Table 3).

**TABLE 3:** Results of the hierarchical multiple linear regression analyses on with total score of the Metacognition Assessment Interview (MAI) as the dependent variable.

Predictors	*B*	*R*^2^	*R*^2^ change	*F* change	*p* (*F* change)
Block 1		0.119	0.119	3.84	0.006
SES	0.11				
MPS	0.03				
NPI	−0.25**				
HSNS	−0.05				
Block 2		0.169	0.050	6.80	0.010
Induction^§^	−1.89*				

SES, Rosenberg Self-Esteem Scale; MPS, Multidimensional Perfectionism Scale; NPI, Narcissistic Personality Inventory; HSNS, Hypersensitive Narcissism Scale; B, unstandardized regression coefficient. *p < 0.05, **p < 0.01, ^§^ 0 = no, 1 = yes.

In order to investigate whether being primed with an experience of failure affected the capacity to understand both ones’ own and others’ mental states, the same analysis was repeated with the MAI Self score and with the MAI Other score as the dependent variables. The trait-like control variables accounted for 7.1% of the variance of Self mindreading and of the 12.9% of the variance of other mindreading, and again overt narcissistic traits showed the greatest effect. However, after controlling for all the baseline variables, the induction accounted for an additional 10.1% of the variance of Self mindreading only, while the effect on Other mindreading was not significant (Table 4). The tolerance values for all the independent variables were larger than 0.67 and the variance inflation factor never exceeded 1.50, suggesting that multicollinearity between our predictor variables was low (Belsley et al., 1980).

**TABLE 4:** Results of the hierarchical multiple linear regression analyses on with Self and Other mindreading as the dependent variables.

Predictors	*B*	*R*^2^	*R*^2^ change	*F* change	*p* (*F* change)
**DV – MAI self**					
Block 1		0.071	0.071	2.00	0.101
SES	0.03				
MPS	0.03				
NPI	−0.11*				
HSNS	−0.05				
Block 2		0.172	0.101	12.69	0.001
Induction^§^	−1.63**				
**DV – Mai other**					
Block 1		0.129	0.129	3.82	0.006
SES	0.09				
MPS	−0.01				
NPI	−0.13**				
HSNS	0.01				
Block 2		0.140	0.011	1.31	0.255
Induction^§^	−0.46				

MAI, Metacognition Assessment Interview; SES, Rosenberg Self-Esteem Scale; MPS, Multidimensional Perfectionism Scale; NPI, Narcissistic Personality Inventory; HSNS, Hypersensitive Narcissism Scale; B, unstandardized regression coefficient. *p < 0.05, **p < 0.01, ^§^ 0 = no, 1 = yes.

## Discussion

We investigated the influence of social rank motive in humans. Our idea was that humans tend to display a reduced capacity to reflect and describe their own mental states and those of others when primed with a sense of failure vs. when they just have to recall past experiences of failure. Our goal was to understand whether actually experiencing failure affected mindreading when participants had to report narratives focused on personal experiences of competition. This is an aspect of mindreading which, to the best of our knowledge, has not been previously investigated.

Our hypothesis was that the capacity to reflect on mental states, or mindreading, would be lower in participants undergoing an induction of failure (online experience of the experimental group), as compared to participants who only had to reflect about a past experience of failure without actually experiencing it (offline experience of the control group). Results supported our hypothesis: inducing a sense of failure lead to lower ability to describe both one’s own and others’ mental states in a semi-structured interview (MAI), and this effect was particularly evident with regard to self-reflection. Our results are consistent with earlier findings which have demonstrated that mindreading capacity is diminished during competitive interactions (Lee et al., 2018). What they further show is that the effect of social rank on mindreading depends on how vividly this social motive is experienced and perceived.

When participants are primed to experiencing failure in the moment, that is failing systematically a game against an opponent, their capacity to form a larger and nuanced understanding of what passes through their mind and in others’ minds is reduced. Why so? It is likely that when they actual fail, their attention is focused on personal flaws or on the idea the others will judge them, and so they neglect other aspects of their subjective experience, such as their vulnerabilities, when faced an on line failure, participants may be driven by defensive purposes, so they may be less prone to disclose a wide range of thoughts and emotions, as they depict the other as superior, spiteful, critical or rejecting. In the same time, when reflecting about the others they may catch a range of thoughts and emotions, though these are likely mostly concerned about how others are evaluating their performance. In other terms, failing a task peculiarly affects capacities for self-reflection and overall understanding of mental states in human interactions, while the specific capacities to understand the others is less impaired, probably because the person is paying much attention to signs of criticism or rejection.

On the other hand, we are not in a position to say whether simply remembering failure was associated to impaired mindreading as we have not normative score for the MAI. In order to better understand if simply focusing on failure has detrimental effects on mindreading, we would have needed to compare this condition with other conditions of success and of being driven by other motives, such as, for example, attachment, sexuality or exploration.

There are different possible explanations for this finding. One is that when people focus on their experience of defeat they lose the capacity to form a bigger picture of what is passing through their own and others’ minds. Their attention is concentrated on personal flaws or on the idea the others will judge them, despise them or reject them, and so they neglect other aspects of their subjective experience, such as their vulnerabilities, their involvement in the task, the relevance of the task for their personal life, or the presence of other aspects of their experience which are not connected to task performance. At the same time, when faced with failure, participants may be driven by defensive purposes, so they may be less prone to disclose a wide range of thoughts and emotions, as they depict the other as superior, spiteful, critical, or rejecting. Overall, these results highlight the importance of taking into account the online interpersonal contex in which mindreading assessment takes place.

We also controlled for potential confounds, as the literature shows that mindreading could be affected by trait-based sensitivity to issues of social rank, e.g., low self-esteem, high maladaptive perfectionism, and both overt and vulnerable narcissism (Kernberg, 1975; Huprich, 2014; Dimaggio et al., 2015a). Our second hypothesis was that trait-based personality dispositions linked to social rank, that is self-esteem, perfectionism and both grandiose and vulnerable narcissism were connected to mindreading. The idea was that persons with low self-esteem, high perfectionism and high grandiose and vulnerable narcissism may have had more reduced mindreading when primed with actual experiences of failure. Our results only supported our hypothesis regarding grandiose narcissism. It appears that persons high in this trait suffers more the effect of experiences of failure. A possible explanation is that these persons resort to grandiosity as a defense against underlying a sense of low personal worth (Kohut, 1971). Therefore, when confronted with a defeat they have not time to deny, they suffer more distress and automatically focus on how to protect themselves against emerging feelings and ideas of shame and inferiority and therefore their capacity to explore the range of psychological experiences passing through the participants in social interactions is reduced. Conversely, absence of influence of other traits seems to speak for the relevance of experiences of failure on human capacity to reason on mental states. We underscore that our was a study based on a non-clinical sample, therefore it is well possible that investigations in participants with significant psychopathology the influence of maladaptive traits of low self-esteem, perfectionism and vulnerable narcissism becomes relevant. Priming with current experiences of failure participants with eating disorders, depression and personality disorders or other conditions, would lead them to express reduced capacities for mindreading.

Even though these trait-based dispositions, especially grandiose narcissisms, affected mindreading, our results show that experiencing failure significantly narrowed the ability to reason about one’s own mind, even after controlling for all these variables.

If supported by future studies, these results may have relevant implications. In the clinical field, it might be necessary to make clients aware of their sensitivity to failure and of how their ability to reflect on mental states might suffer when they are prey to feelings and ideas of failure or criticism or rejection. Particularly in clinical settings and when evaluating psychiatric populations, it is essential to remain aware of the emotions being activated during clinical interactions which may evoke or be associated with competitive experiences. The data emerging from this study underline the importance of ongoing assessment of the patient’s active emotional state and especially whether a competitive mental state is being activated. It also supports the idea that failure in mindreading is not just connected to problems in the attachment domain, but depends also on the activation of social rank motive (Dimaggio et al., 2007, 2015b; Liotti and Gilbert, 2011; Gilbert, 2014; Colle et al., 2017; Popolo et al., 2019). It looks like that actual experiences of failures, something the person experiences in the moment, disrupts capacity to think about mental states, more than just remembering similar experiences. Therefore these data suggest two important point in psychotherapy 1. When dealing with social rank issues, in some patients with higher tendencies to emotion dysregulation, it may be easier to start from remembered episodes of failures when trying to work them through, so their capacity to think about them is more preserved. 2. Our data also support the idea the importance of keeping cooperative motive in psychotherapy and avoiding competitive interaction between the patient and the therapist, in order to make treatment really effective. Our result confirm that the pressure of competition may narrow our capacity to make sense of mental states, so we may have limited abilities in the very moment we need them. Clinical data and observations already highlighted how mindreading abilities are better preserved in cooperative interaction rather than when competitive motive is active in psychotherapy session (Colle et al., 2017; Monticelli et al., 2018; Dimaggio et al., 2020).

Even though our results are promising, there are many limitations which need to be considered. For one, the sample was small, which decreases statistical power and increases the likelihood of a type II error (“false negative” findings or conclusions). In particular, we could expect that with a bigger sample size some trait-based dispositions, such as grandiose and vulnerable narcissism, would become significantly related in the condition of online defeat.

Second, our sample was made up of western Caucasian, mostly well-educated and young participants, therefore our results cannot be generalized to people from other ethnicities, lower levels of education or older age group. Third, many other variables may have influenced results, for example psychological symptoms, tendency to worry, poor emotion regulation, and so forth. Moreover, we did not assess attachment history, so it remains necessary to control for its influence. We assume that social rank may predict mindreading capacity even after controlling for attachment history, as mindreading and attachment are different systems with different evolutionary purposes, but this is a matter for future research. Overall, there is a need to extend our findings controlling for variables we did not consider here. Fourth, our study only explored the effect of experiences of failure. We are not in a position to predict whether the reverse is true, i.e., whether or how experiences of success influence mindreading. All possibilities are open: mindreading capacities may remain unaffected by experiences of success, or people may instead experience a benefit and become more open and capable of reflecting upon their own mental states and those of others; it is also possible that success may have a detrimental effect on mindreading as people may remain entrenched in the social rank motive and lose the capacity to grasp the bigger picture of what they and others may think and feel. Fifth, our was a study with a community sample, so results cannot be generalized to people with mental health problems, such as depression, anxiety, PTSD, or personality disorders. Many have noted that these populations tend to have diminished capacities for mindreading (Lee et al., 2005; Nietlisbach and Maercker, 2009; Hezel and McNally, 2014; Semerari et al., 2014). Mindreading performance of patients with PDs appears to be particularly sensitive to the relational context of interactions so it is relevant to understand under what interpersonal conditions these capacities are more negatively affected.

These limitations notwithstanding, our study yields further support to earlier findings that, when caught by social rank, humans become less able in mindreading and noted how self-reflection is particularly affected. More studies are needed to explore how the social rank motive affects mindreading, under what conditions and in which sub-populations. This would pave the way for a deeper understanding of how humans make decisions under these circumstances and of how to counteract the detrimental effect of experiences of failure.

## Data Availability Statement

The datasets generated for this study are available on request to the corresponding author.

## Ethics Statement

The studies involving human participants were reviewed and approved by Ethics Committee of IUSTO, Università Pontificia Salesiana di Torino, Italy. The patients/participants provided their written informed consent to participate in this study.

## Author Contributions

LC designed the research, analyzed the data, and wrote the manuscript. GD and AS designed the research and wrote the manuscript. AC performed the research. GN analyzed the data and prepared the figures. CC designed the research, recruited the participants, analyzed the data, and wrote the manuscript.

## Conflict of Interest

The authors declare that the research was conducted in the absence of any commercial or financial relationships that could be construed as a potential conflict of interest.
